# Interventional radiology for prevention and management of postpartum haemorrhage: a single centre retrospective cohort study

**DOI:** 10.1007/s00404-024-07595-y

**Published:** 2024-06-16

**Authors:** Jazz Storms, Kristel Van Calsteren, Liesbeth Lewi, Geert Maleux, Johannes van der Merwe

**Affiliations:** 1 Department of Obstetrics and Gynaecology, Division Woman and Child, University Hospitals Leuven, Herestraat 49, 3000 Leuven, Belgium; 2 Department of Radiology, University Hospitals Leuven, Herestraat 49, 3000 Leuven, Belgium

**Keywords:** Obstetric haemorrhage, Postpartum bleeding, Uterine artery embolization, Pelvic artery embolization, Pregnancy

## Abstract

**Purpose:**

Postpartum haemorrhage (PPH) remains a leading cause of maternal death despite current medical management. Surgical interventions are still needed for refractory bleeding. Interventional radiology (IR) can be a successful intermediary that avoids the need for hysterectomy. Nevertheless, IR outcome data in a peripartum setting are limited. The objective of this study is to document the efficacy and safety of IR.

**Methods:**

Retrospective study reviewed the records of consecutive patients who underwent peripartum IR from 01/01/2010 until 31/12/2020 in a tertiary academic centre. Patients were divided in a prophylactic and a therapeutic group. Information about interventions before and after IR, and IR specific complications was retrieved. Efficacy was defined by the number of transfusions and additional surgical interventions needed after IR, and safety was assessed by the incidence of IR related complications.

**Results:**

Fifty-four patients, prophylactic group (*n* = 24) and therapeutic group (*n* = 30), were identified. In both groups, IR was successful with 1.5 ± 2.9 packed cells transfused post-IR (1.0 ± 2.1 prophylactic vs 1.9 ± 3.3 therapeutic; *p* = 0.261). Additional surgical interventions were required in *n* = 5 patients (9.2%), *n* = 1 (4.2%) in the prophylactic vs. *n* = 4 (13.3%) in the therapeutic group. Complications were reported in *n* = 12 patients (22.2%), *n* = 2 (8.3%) prophylactic vs. *n* = 10 (33.3%) in therapeutic group. Mostly minor complications, as puncture site hematoma or bleeding, were reported in *n* = 4 (7.4%). Severe complications as necrosis and metabolic complications were reported in *n* = 2 patients (3.9%).

**Conclusion:**

IR for prevention and treatment of PPH was highly successful and associated with minor complications.

## What does this study add to the clinical work

**Table:** 

Interventional radiology (IR) for prevention and treatment of postpartum haemorrhage (PPH) is effective and safe, with mostly minor complications. Future research is needed to determine the impact of IR in the peripartum setting on subsequent fertility and pregnancy outcomes.	

## Introduction

Prevalence of postpartum haemorrhage (PPH) is between 1 and 6% of deliveries with a wide variation amongst different regions in the world [1, 2]. In Belgium, B.OSS collected all cases of PPH requiring hysterectomy or management by IR between January 2012 and December 2013 and estimated the prevalence of major PPH at 6.6 in 10 000 deliveries [3].

PPH is defined by a blood loss of 1000 ml or greater, or blood loss with associated signs or symptoms of hypovolemia, regardless of the mode of delivery [4]. PPH is the leading direct cause of maternal death with a 1% mortality [5, 6]. Beyond the mortality risk, PPH more often leads to maternal morbidity such as hypovolemic shock, diffuse intravascular coagulopathy, respiratory distress syndrome and kidney- and liver failure [7]. The most common cause of PPH, is uterine atony followed by placenta retention, coagulopathy, or trauma to the genital tract [7, 8]. Currently, standard of care entails *oxytocin* administration during the third stage of labour [9]. However, in 2% of births, additional interventions are needed to address ongoing PPH, e.g. medical options, such as *ergot alkaloids* and *prostaglandins* [10]. While refractory bleeding may require surgical interventions like uterine compression sutures, uterine artery vessel ligation, and hysterectomy [11]. Next to surgical interventions, interventional radiology (IR) can also be a successful strategy for refractory PPH and avoid the need for a hysterectomy.

IR has a success rate between 79 and 95% for refractory PPH [11–14]. Belgian data from 2013 showed that 50% of the 166 refractory PPH cases were managed by hysterectomy, and only 39% by IR only [3]. Furthermore, IR appears to be an excellent intermediary management choice if there is a future fertility wish since limited evidence suggests that IR does not affect future fertility or pregnancy rates [15]. Nevertheless, IR carries an inherent procedural risk of 6–7%, with post-embolization fever, hematoma at the puncture site, infections, and (transient) ischemia [16, 17]. Rarely, even more severe complications like uterine infarction [12] and thrombo-embolic events have been reported [17].

In view of the limited outcome data of IR for the prevention or management of PPH, this retrospective study aimed to review the efficacy and safety of peripartum IR in a tertiary care setting. We also compare the outcomes of IR between patients who underwent IR as a preventative measure (prophylactic) and those who underwent IR as a measure to address refractory PPH (therapeutic).

## Methods

### Aims

Our primary aim was to investigate and compare the efficacy and safety of prophylactic and therapeutic IR. Efficacy was assessed by analysing the total number of transfusions and surgical interventions done post-IR. Safety was assessed by reviewing the IR related complications, such as post-embolization fever, hematoma at the puncture site, thrombo-embolic events, necrosis and aneurysms.

Our secondary aim was to review the maternal morbidity of these women that included persistent vegetative state defined as a state of wakefulness without awareness, cerebrovascular accident defined as on medical imaging confirmed cerebrovascular accident, pulmonary oedema defined as radiographic evidence of pleural effusion, Mendleson’s syndrome aspiration syndrome resulting in chemical pneumonitis, kidney failure as defined by the RIFLE criteria [18], thrombotic event defined as any form of thromboembolic event in the 6 weeks postpartum, septicaemia defined as any form of sepsis in the 6 weeks postpartum. required ventilation defined as the need for invasive mechanical ventilation despite supplemental oxygen therapy, disseminated intravascular coagulation (DIC) based on laboratory findings that indicate activation of the coagulation cascade, liver failure as defined by the MELD score [19], bladder or ureter injury defined as urinary tract disruption or leaking, confirmed on medical imaging, and bowel dysfunction such as prolonged ileus, based on clinical or radiologic findings.

### Patients and setting

This single centre retrospective cohort study reviewed the records of all patients who underwent IR at time of birth from 01/01/2010 until 31/12/2020 at the University Hospital of Leuven, Belgium. This study was approved by the local Ethical Committee (MP017199). Peripartum period was defined as immediately before birth or within 6 weeks postpartum. Data were collected from medical files. Supplementary file 1 shows the list of all variables that were retrieved.

Peripartum patients were subdivided into two groups; prophylactic balloon occlusion to prevent PPH (prophylactic group) and therapeutic embolization (therapeutic group) for PPH. Patients were further subdivided by the specific IR technique used: (1) uterine artery embolization, (2) internal iliac artery embolization, (3) multiple pelvic artery embolization, (4) uterine artery or internal iliac artery balloon occlusion and (5) aorta balloon occlusion.

### Local clinical guidelines/protocols and definitions

Prophylactic IR by balloon occlusion prior to delivery was mainly done in cases where placenta accreta spectrum (PAS) disorder was suspected or when a caesarean–hysterectomy was anticipated but also in some cases when otherwise, there was a massive obstetric haemorrhage risk. Therapeutic embolization was mainly used to cease refractory PPH when medical and mechanical treatment, with an intra-uterine occlusion balloon, was insufficient.

Definitions for placenta previa and PAS were used according to the RCOG [20]. Suspected PAS was defined as an abnormally adherent or invasive placenta suspected on transabdominal or transvaginal ultrasound by a trained sonographer.

### Statistics

Statistical data analysis was performed using IBM SPSS Statistics 28.0 (SPSS Inc, Chicago, IL). Continuous data were tested for normality using the Shapiro–Wilk tests and reported as mean and standard deviation or median with interquartile range, as appropriate. Differences between continuous variables were tested by independent samples *t* tests or Mann–Whitney *U* tests. Categorical data were reported as number per group and percentage. *χ*^2^ or Fisher's exact test were used for comparison of categorical variables. A two-sided *p* value of < 0.05 was considered significant.

## Results

Between 01/01/2010 and 31/12/2020, 54 women underwent IR in a peripartum setting, of which, 24 patients underwent a prophylactic balloon occlusion and 30 patients received a therapeutic embolization for refractory PPH. In one case, a prophylactic balloon occlusion was followed by a therapeutic embolization for refractory PPH. This case was categorized in the prophylactic group.

Table 1 summarizes the baseline characteristics of the included patients.

**Table 1:** Baseline characteristics

	Total (*n* = 54)	Prophylactic (*n* = 24)	Therapeutic (*n* = 30)	*p* value
Caucasian	72.2% (39/54)	62.5% (15/24)	80% (24/30)	0.041
Age	33.7 ± 5.2	34.3 (5.6)	33.6 (5.0)	0.632
BMI	21.5 (4.2)	24.0 (5.3)	21.7 (2.8)	0.067
Smoking (any)	7.4% (4/54)	12.5% (3/24)	3.3% (1/30)	0.449
Recreational drug use (any)	1.9% (1/54)	4.2% (1/24)	0% (0/30)	0.259
Pre-existing medical conditions	40.7% (22/54)	50% (12/24)	33.3% (10/30)	0.301
Primigravida	22.2% (12/54)	8.3% (2/24)	33.3% (10/30)	0.028
Previous vaginal birth	29.6% (16/54)	16.7% (4/24)	40% (12/30)	0.167
Previous caesarean birth	40.7% (22/54)	90.9% (20/24)	6.7% (2/30)	< 0.001
Singleton pregnancy	92.6% (50/54)	91.7 (22/24)	93.3% (28/30)	0.816

*p < 0.05. Categoric parameters are expressed as n and (%) of total within group. Continuous parameters are expressed as mean ± SD

*BMI* body mass index. Possible medical conditions were asthma, epilepsy, psychiatric, endocrine, haematological, autoimmune, and cardiac disorders, endometriosis, fibromas, and history of molar pregnancy

Uterine abnormalities were described in 3 out of 54 cases, 1 uterus subseptus and 2 cases of uterine fibromatosis. All three of them underwent therapeutic embolization for PPH.

Out of the total group, *n* = 22 (40.7%) of patients who had a previous caesarean section, *n* = 12 (22.2%) patients had two previous caesarean sections, *n* = 4 (7.4%) had three previous caesarean sections and *n* = 2 (3.7%) had four previous caesarean deliveries. All the cases with more than one previous caesarean deliveries underwent prophylactic IR.

The characteristics and the general outcomes of the index pregnancy are represented in Table 2. The most common obstetric complications, apart from placenta previa and placenta accreta spectrum disorder (PAS), were pre-eclampsia in *n* = 5 patients (9.3%) (*n* = 0 (0%) in the prophylactic group vs. *n* = 5 (16.7%) in the therapeutic group, *p* = 0.059), preterm labour in *n* = 2 patients (3.7%) (*n* = 1 (4,2%) in the prophylactic group vs. *n* = 1 (3.3%) in the therapeutic group, *p* = 0.696), gestational diabetes in *n* = 2 patients (3.7%) (*n* = 1 (4.2%) in the prophylactic group vs. *n* = 1 (3.3%) in the therapeutic group, *p* = 0.696), and premature rupture of membranes (PROM) in *n* = 2 patients (3.7%) (*n* = 2 (8.3%) in the prophylactic group vs. *n* = 0 (0%) in the therapeutic group, *p* = 0.193). Placenta previa was noted in *n* = 21 patients (38.9%), while PAS was suspected in *n* = 20 (37.1%) patients. Most patients were delivered by caesarean section in this cohort.

**Table 2:** Characteristics of the pregnancy and general outcomes

	Total (*n* = 54)	Prophylactic (*n* = 24)	Therapeutic (*n* = 30)	*p* value
Placenta previa I	9.3% (5/54)	–	17.2% (5/30)	0.058
Placenta previa II	5.6% (3/54)	12.5% (3/24)	–	0.082
Placenta previa III	1.9% (1/54)	4.2% (1/24)	–	0.444
Placenta previa IV	22.2% (12/54)	50% (12/24)	–	< 0.001
Any antepartum bleeding	27.8% (15/54)	41.7% (10/24)	17.2% (5/30)	0.041
PAS suspected	37.1% (20/54)	75% (18/24)	6.7% (2/30)	< 0.001
Planned vaginal delivery	51.9% (28/54)	8.3% (2/24)	86.7% (26/30)	< 0.001
Caesarean delivery	64.8% (35/54)	100% (24/24)	36.7% (11/30)	< 0.001
Delivery GA > 37 weeks	44.4% (24/54)	20.8% (5/24)	63.3% (19/30)	0.001
Delivery GA < 37–34 weeks	33.3% (18/54)	37.5% (9/24)	30% (9/30)	0.772
Delivery GA < 34–28 weeks	20.4% (11/54)	37.5% (9/24)	6.6% (2/30)	0.007
Delivery GA < 28 weeks	1.9% (1/54)	4.2% (1/24)	–	0.444
Intentional placenta retention	29.6% (16/54)	37.5% (9/24)	23.3% (7/30)	0.369
Planned caesarean hysterectomy	16.7% (9/54)	37.5% (9/24)	–	< 0.001
Unplanned caesarean hysterectomy	9.3% (5/54)	8.3% (2/24)	10% (3/30)	0.607
PAS confirmed on pathology	9.3% (5/54)	20% (5/24)	–	0.013
Bladder or ureter injury	7.4% (4/54)	16.7% (4/24)	–	0.034
Invasive ventilation	5.6% (3/54)	–	10% (3/30)	0.245
Bowel dysfunction	1.9% (1/54)	–	3.3% (1/30)	1.0
Kidney failure	7.4% (4/54)	4.2% (1/24)	10% (3/30)	0.620
Pulmonary oedema	5.6% (3/54)	–	10% (3/30)	0.245
DIC	3.7% (2/54)	4.2% (1/24)	3.3% (1/30)	0.872
Septicaemia	14.8% (8/54)	–	26.7% (8/30)	0.006
Maternal mortality	–	–	–	–
HDU/ICU admission	27.8% (15/54)	16.7% (4/24)	36.7% (11/30)	0.112
5 min Apgar	9.0 (2.7)	7.5 (2.4)	8.1 (3.1)	0.431
Neonate survival at discharge	96.3% (52/54)	95.8% (23/24)	96.7% (29/30)	0.087
Neonatal morbidity	38.9% (21/54)	66.7% (16/24)	16.7% (5/30)	< 0.001
NICU admission	63% (34/54)	75% (18/24)	53.3% (16/30)	0.156
Neonatal mortality	3.7% (2/54)	4.2% (1/24)	3.3% (1/30)	0.872

*p < 0.05. Categoric parameters are expressed as n and (%) of total within group. Continuous parameters are expressed as mean ± SD

*PAS* placenta accrete spectrum, *GA* gestational age. Possible neonatal complications were respiratory distress syndrome, necrotising enterocolitis, chronic lung disease, severe jaundice requiring phototherapy, severe infection e.g. septicaemia, meningitis, and exchange transfusion. Pathology was only performed on 19 of the 24 placentas of the prophylactic group and pathology was not performed in the therapeutic group

The average delivery gestation was 36w5d ± 25 days (Prophylactic 34w0d ± 21 days vs. PPH 38w3d ± 18 days).

All nine planned caesarean–hysterectomies were for suspected PAS, and in *n* = 5 patients (55.5%) PAS was confirmed by histopathology afterwards. Another *n* = 5 cases (9.3%) underwent an unplanned caesarean-hysterectomy due to failed expectant management for a suspected PAS (*n* = 1), a placenta previa (*n* = 1), refractory PPH (*n* = 2) and in one case, a subtotal hysterectomy was performed on day 26 (*n* = 1). This last case was delivered vaginally at term but due to refractory PPH required uterine artery embolization with subsequent septic necrosis of a multi-fibroid uterus.

The medical and surgical interventions before and after IR are listed in Table 3. As expected in the therapeutic group, the main intervention before IR was the administration of uterotonics and clotting factors. In this group the average blood loss before IR was already in excess of 2 L. In the therapeutic group, *n* = 4 patients (13.3%) required additional surgical interventions, namely three brace sutures (two before IR and one after IR) and one uterine artery ligation before IR. In the prophylactic group, one case required an additional brace suture after IR.

**Table 3:** Efficacy of prophylactic and therapeutic IR

	Total (*n* = 54)	Prophylactic (*n* = 24)	Therapeutic (*n* = 30)	*p* value
Interventions before IR				
* Oxytocin* and/or *Carbetocin*	55.6% (30/54)	–	100% (30/30)	< 0.001
Other uterotonics	18.5% (10/54)	–	33.3% (10/30)	0.003
Clotting factors	35.2% (19/54)	–	63.3% (19/30)	< 0.001
Packed red blood cells	2.5 (3.4)	0.3 (1.4)	4.4 (3.4)	< 0.001
FFP	1.5 (2.5)	0.3 (1.2)	2.5 (2.9)	0.002
Platelets	0.6 (1.1)	–	0.6 (1.0)	–
Intra-uterine balloon	14.8% (8/54)	–	26.7% (8/30)	0.399
EBL (ml) pre-IR	1382 (1659)	–	2598 (1409)	–
Interventions after IR				
* Oxytocin* and/or *Carbetocin*	70.4% (38/54)	87.5% (21/24)	56.7% (17/30)	0.014
Other uterotonics	38.9% (21/54)	30.4% (7/24)	46.7 (14/30)	0.281
Clotting factors	22.2% (12/54)	33.3% (8/24)	13.3% (4/30)	0.064
Packed red blood cells	1.5 (2.9)	1.0 (2.1)	1.9 (3.3)	0.261
FFP	1.1 (2.8)	0.7 (1.7)	1.3 (3.5)	0.461
Platelets	0.4 (0.7)	0.2 (0.5)	0.5 (0.8)	0.183
Intra-Uterine Balloon	14.8% (8/54)	4.2% (1/24)	23.3% (7/30)	0.086
EBL (ml) post-IR	805 (1149)	1321 (1005)	417 (1112)	0.005

*p < 0.05. Categoric parameters are expressed as n and (%) of total within group. Continuous parameters are expressed as mean ± SD. Other uterotonics contain ergometrine, prostaglandins, etc. Clotting factors contain fibrinogen, recombinant factor VII, tranexamic acid etc

*FFP* fresh frozen plasma, *EBL* estimated blood loss, *IR* interventional radiology

The complications per specific IR technique are depicted in Table 4. Overall, *n* = 12 patients (22.2%) had one or more complication. No difference was seen between the two main groups (Prophylactic 8.3% (2/24) vs. PPH group 33.3% (10/30), *p* = 0.0748). The most common complication was a haematoma or bleeding at the puncture site with *n* = 4 patients (7.4%). The most severe complication was uterine necrosis in *n* = 2 patients (3.7%). One of these cases was managed by antibiotics and the other required a delayed hysterectomy, as mentioned above. One patient developed a severe hyperkalaemia and kidney failure due to haemolysis post embolization.

**Table 4:** Safety outcomes of IR procedures

	Total (*n* = 54)	Therapeutic			Prophylactic	
		Uterine artery embolization (*n* = 22)	Internal iliac artery embolization (*n* = 1)	Multiple arteries embolization (*n* = 7)	Uterine artery or internal iliac artery occlusion (*n* = 22)	Aorta occlusion (*n* = 2)
Uncomplicated	81.5% (44/54)	72.7% (16/22)	100% (1/1)	57.1% (4/7)	90.9% (20/22)	100% (2/2)
Haematoma/haemorrhage	7.4% (4/54)	9.1% (2/22)	–	14.3% (1/7)	4.5% (1/22)	–
Sepsis/fever	1.9% (1/54)	4.5% (1/22)	–	–	–	–
VTE	1.9% (1/54)	4.5% (1/22)	–	–	–	–
Necrosis	3.7% (2/54)	9.1% (2/22)	–	–	–	–
Aneurysm	1.9% (1/54)	–	–	–	4.5% (1/22)	–
Metabolic	1.9% (1/54)	–	–	16.7% (1/6)	–	–

*p < 0.05. Categoric parameters are expressed as n and (%) of total within group

## Discussion

In our study, we encountered 54 patients that underwent IR in a peripartum setting over a time span of 10 years. In most cases, IR was a successful adjunct in the management of refractory PPH when reflected on the transfusion need of only 1.5 ± 2.9 units of packed red blood cells after IR. Furthermore, the need for surgical interventions after IR was low with 9% of the cases requiring an unplanned hysterectomy mostly due to uterine atony or failed expectant management of suspected PAS. However, in about one in five of the cases, an IR related complication was encountered, though only 4% was considered severe, including uterine necrosis (*n* = 2) or metabolic perturbations, such as hyperkalaemia.

In 90% (27/30) of the refractory PPH cases, no additional surgical interventions were needed and 88% (21/24) of the prophylactic cases did not acquire any additional procedures. These findings are in line with the findings of a systematic review by Mei et al. [21] where a success rate, defined as no need for subsequent surgical interventions, of 89.8% and 78.6% was found for artery embolization and balloon occlusion respectively. In this review, around 11 to 30% of the cases underwent a secondary hysterectomy. Additionally, the Belgian, B.O.SS registry, also reported on a success rate, defined as no need for subsequent surgical interventions, of 88% [3], reflecting the expected contemporary outcomes to be expected.

In 90% of cases in the prophylactic setting, placenta previa was diagnosed or PAS was suspected. As diagnosis of these complications is set before delivery, an elective caesarean section is planned and FIGO recommendations on PPH, advise to consider balloon occlusion when excessive blood loss is expected [22, 23]. In our study, a prophylactic balloon occlusion was considered effective given the estimated blood loss (EBL) after IR was an average of approximately 417 ± 1112 ml and an average of 1.9 ± 3.3 units packed red blood cells was needed. A case series of Sadashivaiah et al., showed similar results with an EBL of 800 ml and a transfusion need of 1 to 4 units packed red blood cells after IR [24].

The most common complication reported in our study was haematoma or bleeding of the puncture site with an incidence of 7.4% which seems lower than reported in other reports where the incidence is estimated at 11–13% [25–27]. Due to the retrospective character of our study, minor haematomas might not have been reported. In a large prospective study about access site complications after endovascular procedures, the incidence of haematomas was estimated 13% [27]. Remarkably, a higher incidence was reported in female patients. A possible explanation was a smaller vessel diameter and a mismatch with the larger sheath size used. Another explanation was a lower bodyweight compared to man and, therefore, a relatively high dose of heparin used for the procedure [27]. This highlights the need for more dedicated research in female, especially pregnant, patients undergoing IR.

The most severe complication reported in our study was uterine necrosis, requiring a delayed hysterectomy. This is a rare but life-threatening complication that is reported in only a few cases after uterine artery embolization for the treatment of symptomatic myomas. The aim of this procedure is to interrupt the uterine blood supply and cause necrosis and ischemia of the myoma which will lead to shrinking of the myoma [28, 29]. However, necrosis of the myoma can cause ischemic injuries and can ultimately lead to secondary sepsis [30]. About 20 cases of uterine necrosis have been reported in literature [31, 32]. In the majority of these cases, adjunct surgical management, myomectomy or hysterectomy, was required. This questions the role of IR in the management of refractory PPH in patients with a uterus myomatosus.

This comprehensive single institution review over a 10-year period collected a large amount of data and outcome measures about efficiency as well as safety. With these results, the study contributes to the existing literature about IR for management of PPH. As for Belgium, in comparison with the B.O.SS study [3], we describe a more detailed picture of efficacy and safety of IR in an obstetric setting. This study encountered some obvious limitation due to the retrospective character of the study and the limited number of patients. Especially, the lack of significance could be due to the small study group. A prospective database will be required to evaluate the efficacy and safety of IR in peripartum setting. Also, we did not investigate the impact of IR on subsequent fertility and pregnancy outcomes after IR. In general, there is limited data about fertility after embolization for obstetric reasons, but normal menses and pregnancies have been reported after IR for PPH [33, 34] Results from a retrospective cohort study in our own centre, showed 40% pregnancy rates after embolization for uterine fibroids [35]. High-quality observational data could help to address this gap in knowledge.

In conclusion, we could recommend that in view of the high efficacy and safety of IR, IR could be preferred above surgical management for women at the highest risk of PPH. However, the implantation of IR across all settings is greatly restricted due to the limited availability and technical expertise needed for IR.

## Conclusion

In our population, IR for prevention and treatment of PPH was effective and mostly minor complications were reported. Future research should focus on the impact of IR in the peripartum setting on subsequent fertility and pregnancy outcomes.

## Data Availability

Not applicable.
